# Evaluation of a group based cognitive behavioural therapy programme for menstrual pain management in young women with intellectual disabilities: protocol for a mixed methods controlled clinical trial

**DOI:** 10.1186/1472-6874-14-107

**Published:** 2014-09-08

**Authors:** Susan Kennedy, Siobhan O’Higgins, Kiran Sarma, Carla Willig, Brian E McGuire

**Affiliations:** 1 Brothers of Charity Services, Galway, Ireland; 2 Department of Psychology, City University London, London, UK; 3 School of Psychology, National University of Ireland, Galway, Ireland; 4 Centre for Pain Research, National University of Ireland, Galway, Ireland

**Keywords:** Menstrual pain management, Cognitive behavioural therapy, Intellectual disability

## Abstract

**Background:**

Menstrual pain which is severe enough to impact on daily activities is very common amongst menstruating females. Research suggests that menstrual pain which impacts on daily functioning may be even more prevalent amongst those with intellectual disabilities. Despite this, little research attention has focused on pain management programmes for those with intellectual disabilities.

The aims of this pilot study were to develop and evaluate a theory-based cognitive behavioural therapy (CBT) programme for menstrual pain management in young women with intellectual disabilities.

**Methods/Design:**

The study utilised a mixed methods controlled clinical trial to evaluate elements from a CBT programme called *Feeling Better* (McGuire & McManus, 2010). The Feeling Better programme is a modular, manualised intervention designed for people with an intellectual disability and their carers. The programme was delivered to 36 young women aged 12 – 30 years who have a Mild - Moderate Intellectual Disability, split between two conditions. The treatment group received the *Feeling Better* intervention and the control group received treatment as usual. To evaluate the effectiveness of the programme, measures were taken of key pain variables including impact, knowledge, self-efficacy and coping. Process evaluation was conducted to examine which elements of the programme were most successful in promoting change.

**Discussion:**

Participants in the intervention group were expected to report the use of a greater number of coping strategies and have greater knowledge of pain management strategies following participation in the intervention and at three month follow-up, when compared to control group participants. A significant advantage of the study was the use of mixed methods and inclusion of process evaluation to determine which elements of a cognitive behavioural therapy programme work best for individuals with intellectual disabilities.

**Trial registration:**

Current Controlled Trials ISRCTN75567759

## Background

The International Association for the Study of Pain (IASP) defines pain as “an unpleasant sensory and emotional experience associated with actual or potential tissue damage, or described in terms of such damage” [1]. Although definitions of what constitutes “chronic” pain vary, the IASP definition of pain lasting more than 3 months is widely accepted. Walsh, Morrison and McGuire [2] examined chronic pain in adults with an intellectual disability and found that chronic pain was experienced by 15% of adults with an intellectual disability, based on caregiver report. Whilst this is consistent with reports of the frequency of chronic pain in the general population, it has been suggested that this may be an under-estimate of the extent of the issue in those with intellectual disabilities, especially amongst those who are non-verbal or have a more severe level of disability [3]. As those with more severe intellectual disabilities are not always able to verbally communicate their pain to carers, their pain experience may not always be recognised and reported. Whilst the use of proxy respondents can also be beneficial in gathering information about the pain experience of those with significant intellectual disabilities and communication challenges, this method presents its own challenges including the issue of reliability of carer report. Other methods such as structured behavioural observation offer a reliable and valid alternative [4] and the use of more than one source of information increases the reliability of the information obtained.

Dysmenorrhea (menstrual pain) is extremely common with as many as 90% of menstruating female adolescents and 50% of women reporting that they suffer from it [5,6]. Kyrkou [7] examined how menstrual pain presents in women with intellectual disabilities as there is anecdotal evidence of an increase in this condition in this population but little research has been conducted in this area. The parents of 24 women with Down Syndrome or Autism Spectrum Disorder (ASD) were surveyed to ascertain how menstrual pain presents in women with intellectual disabilities. Results suggested that two thirds of the women with Autism, three quarters of the women with Down Syndrome and all of the women with Aspergers Syndrome appeared to have problematic period pain. These rates were higher than the 50% rate reported for women in the general population [6] however, due to the small sample size used in the study, caution is required in the interpretation of these findings.

Dysmenorrhea, defined as pain during menstruation which is severe enough to impact or interfere with daily activities [8] has recently been the focus of brain-imaging studies which have shown that the brains of otherwise healthy women with moderate-to-severe dysmenorrhea show significant differences in brain structure and function, when compared with non-dysmenorrheic women. Tu, Niddam and colleagues [9-11] identified differences in cerebral metabolism and cerebral structure for the trait of dysmenorrhea and between pain and pain-free states. Differences have also been found in neural activity induced by noxious skin stimulation when applied to areas remote from the pelvic/abdominal region, such as the arm [12]. An important aspect of these brain-imaging studies is that some of the differences in neural characteristics occurred chronically, throughout the menstrual cycle, even when dysmenorrheic women are not experiencing menstrual pain [13]. Berkley [14] suggests that the consistency of these findings along with those from individuals with other chronic pain conditions provides a strong argument that dysmenorrhea should be considered a chronic pain condition.

Given the potential for significant personal, social and economic impact from chronic pain, much research attention has been directed towards pain management and treatment options. In a review of psychological therapies for the management of chronic pain in the general population, Eccleston, Williams and Morley [15] found that CBT results in improvements in overall functioning and psychological wellbeing. There is also evidence of the effectiveness of such approaches for the treatment of dysmenorrhea [16].

There has been increasing interest in adapting CBT for use with people with an intellectual disability and a few authors have described the use of modified CBT. McCabe [17] described the effectiveness of treatment programmes for depression among adults with mild-moderate intellectual disabilities while Lindsay, Neilson and Laurensen [18] were successful in using this approach to treat anxiety in this population. Willner, Jones, Tam and Green [19] examined the efficacy of a cognitive-behavioural anger management group for individuals with an intellectual disability using a randomized controlled trial research design. They found that clients in the treated group improved on both self- and carer-ratings, relative to their own pre-treatment scores, and relative to the control group post-treatment. Individuals in the intervention group also showed further improvement relative to their own pre-treatment scores, at 3 month follow-up. McGuire and Kennedy [4] noted that there has been limited research evaluating CBT for chronic pain in people with an intellectual disability. An important advance in the area was the development of “*Feeling Better* – a manual for carers working with people who have intellectual disabilities and chronic pain” [20]. This modularised programme uses cognitive behavioural principles to teach individuals with intellectual disabilities a range of strategies to manage chronic pain more effectively. In a case series study by McManus and McGuire [21], some preliminary evidence was provided for the effectiveness of the programme with increases in participant scores on pain management knowledge, wellness-focused coping and effectiveness of coping following the intervention.

In view of the ample evidence that CBT can be used for chronic pain management including the management of dysmenorrhea in the general population, and the preliminary evidence for effectiveness in people with intellectual disability [21], there is a rationale for evaluating a CBT-based pain management programme for menstrual pain in women with an intellectual disability. This study will be the first controlled clinical trial to address the issue of menstrual pain management with individuals with intellectual disabilities. Research on pain in individuals with intellectual disabilities has largely focused on identification of pain and medical management of pain symptoms. Pain management has largely been ignored and pain management programmes have not routinely been offered to such individuals.

### Study aims & objectives

This pilot study aimed to evaluate if a theory-based cognitive-behavioural therapy programme for menstrual pain management (derived from the *Feeling Better* manual) is more effective than usual care in helping young women with intellectual disabilities to manage their menstrual pain. In order to answer this question, a small non-randomised controlled trial was conducted comparing the use of menstrual pain management programme with usual care in an intellectual disability service in the west of Ireland. The study included a number of potential secondary outcome measures in addition to the primary outcome measures of pain coping strategies used and pain knowledge. It will enable us to determine if:

1. Participation in the menstrual pain management group results in an increase in participants’ ratings of pain coping strategies, pain management knowledge and pain self-efficacy and if this is maintained at 3 month follow-up.

2. Participation in the menstrual pain management group results in a reduction in self-ratings of pain intensity and pain interference by participants and reduced ratings of pain intensity and pain interference amongst participants, as rated by their parents, and if these reductions are maintained at 3 month follow-up.

3. Participants whose parents score highly on pain-catastrophizing experience greater pain intensity and greater pain interference with quality of life. Catastrophizing – experiencing extremely negative thoughts about one’s plight and interpreting even minor problems as major catastrophes – appears to be a powerful way of thinking that greatly influences pain and disability and is important in determining one’s reaction to pain [22]. Goubert, Eccleston, Vervoort, Jordan and Crombez [23] found that parents’ catastrophic thinking about their child’s pain had a significant contribution in explaining the child’s disability and school attendance. For this reason, parental catastrophizing is expected to affect participants’ ratings of pain intensity, pain interference and pain coping strategies used.

4. Participants in the menstrual pain management group adopt more behavioural than cognitive coping strategies to manage their menstrual pain. Burkitt et al. [24] found that greater developmental level (rather than chronological age) was associated with the use of more cognitively demanding strategies as may be used in cognitive behavioural therapy treatment approaches. McManus and McGuire [21] also reported a marked absence of the use of cognitive coping skills. Participants were more likely to use behavioural strategies such as exercise and relaxation.

Process evaluation was conducted via qualitative focus groups with participants, parents and support staff. The purpose of this was to assess the acceptability of the intervention to participants, to explore their experiences including any suggestions they may have to further enhance the programme and to examine which elements of the programme were most successful in promoting change for young women with intellectual disabilities who experience menstrual pain. It was envisaged that this innovative approach would yield valuable information which could form the basis for effective interventions to enrich the quality of life of individuals with intellectual disabilities who experience menstrual pain, as well as enhancing the lives of their carers.

## Methods/Design

### Research design

This was a mixed methods study involving both quantitative and qualitative analysis. The sample size of N = 36 was achieved by delivering the pain management programme to three groups of participants in the intervention condition (18 participants in total). A comparison group contained another 18 participants with similar characteristics in terms of age, gender and level of cognitive ability. These individuals received treatment as usual during the study. This typically involved rest and medication, as required. On completion of the study, they will be offered the intervention.

The study design and methodology, based on the Medical Research Council’s (MRC) Framework for Evaluating Complex Interventions [25], was considered an exploratory clinical trial.

### Recruitment & eligibility

#### Setting

This study took place within Galway city and county, in the Republic of Ireland. Recruitment, data collection, intervention and trial management all took place within this region and were coordinated by the primary researcher under the joint supervision of the School of Arts and Social Sciences at City University London and the School of Psychology at the National University of Ireland, Galway (NUIG).

#### Participants

Participants were females with a diagnosis of a mild or moderate intellectual disability who receive support services from the Brothers of Charity Services, Galway. This organisation provides day programmes, residential and respite services, family and multi-disciplinary supports to individuals with intellectual disabilities and to their families. The Diagnostic and Statistical Manual (DSM) framework was used for the diagnoses of intellectual disability and participants IQ scores were in the range 35 – 70.

#### Recruitment strategy

Potential participants who met the inclusion criteria for the study were identified by the Team Leaders for school age and adult services, who have access to such information.

#### Inclusion criteria

Research study participants were females aged between 12 and 30 years of age who have been formally diagnosed using standardized measures of cognitive ability and found to be functioning in the mild or moderate range of intellectual disability. The upper age limit of 30 years was selected to avoid overlap with early menopausal symptoms, as per Kyrkou [7]. Speech was the primary means of communication of research participants. The level of speech of all participants was consistent with their level of intellectual ability, or greater.

Participants were in education or training, attending either a secondary school or an adult training programme. They had commenced menstruation and experienced recurrent pain symptoms with menstruation which impacted on daily functioning.

#### Exclusion criteria

Females were not eligible to participate in the study if they did not have an intellectual disability. Research indicates that cognitive-behavioural strategies may be suitable for individuals with mild and moderate intellectual disabilities and for this reason, individuals with more significant degrees of cognitive impairment were excluded from the study as they would not be able, cognitively, to participate.

In addition, females were not eligible to participate in the study if they were younger than 12 years of age or over 30 years of age, if they had not commenced menstruation, did not experience menstrual pain or if their primary method of communication was via non-verbal strategies.

Participants were not excluded from participating in this research on the basis of ethnicity, race, sexuality, religion or any other socio-cultural factors.

#### Consent

The parents/guardians of potential participants were approached via a participant information letter and asked if they wished to take part in the study and if they consented to their daughter participating in the research. A consent form was provided for this purpose. Once consent was obtained from parents/guardians, assent to participate in the research study was also sought from the young women in question, via a visual participant information sheet and consent form. A copy of these forms was provided to each potential participant and read out to them by the Researcher, at the same time.

### Treatment allocation and matching process

#### Intervention condition

Due to the logistics and practicalities of delivering an intervention group to individuals within a wide geographical sampling area, a non-randomised process was used to assign participants to the intervention group.

A list was compiled of all females attending a special class for students with a mild or moderate intellectual disability or a school for students with mild or moderate intellectual disabilities, who receive support services from the Brothers of Charity Services within County Galway. The Principals of five schools were contacted, informed of the research study and invited to participate in the study. Two schools agreed to participate and were assigned to the intervention group. Parents/Guardians of the relevant students were then contacted and asked for their consent for their daughters to participate. Once consent was granted, assent was obtained directly from the young women.

This treatment allocation methodology allowed for the delivery of the intervention during the school day, at the location where the young women received their day service. This minimised inconvenience and school absence for research participants. This approach also enabled the intervention to be delivered to participants at an appropriate space within the school timetable e.g. during Social, Personal and Health Education (SPHE) class and so ensured consistent attendance.

The same approach was used to recruit participants attending Adult Day Centres providing educational and training opportunities to young women with mild and moderate intellectual disabilities, who receive support services from the Brothers of Charity Services within County Galway.

#### Control condition

Individuals in the control condition were an equivalent comparison group matched by gender, age range and level of intellectual disability. These individuals were invited to participate in the study and were informed that they were allocated to the control condition and what that meant. They were informed that they would be offered the intervention condition, once the study was completed.

### The intervention

#### Programme development

Prior to the main intervention, qualitative preparatory work was completed. Parents/Guardians were invited to take part in a focus group to inform the development of the intervention to best meet the needs of this group of participants. The Participative Research Process (PRP) methodology was chosen for this aspect of the study so that the parents could present their ideas in a “more reflexive, interactive and flexible framework” [26]. The PRP methodology facilitates participants to present their perspectives without filtering or censure by researchers. The PRP allows varying and sometimes unexpected perspectives to emerge as participants create, collate and present their own data. Participants were provided with session outlines of the “*Feeling Better*” programme and asked to consider what should and should not be included in a menstrual pain management programme suitable for their daughters and how this could be done. Parents were asked to respond to a single question: “If your daughter takes part in this group, what would it need to have to help her to cope with menstrual pain?” They then created a ‘web of ideas’ of what they felt was suitable content and delivery methods.

#### Intervention programme

The menstrual pain management intervention programme consisted of twelve sessions:

• Session 1: Psycho-education

• Session 2 – 4: Deep breathing, progressive muscular relaxation and guided visualisation

• Session 5: Taking exercise

• Session 6: Distraction techniques

• Session 7 – 9: How your thoughts make you feel, challenging negative thoughts and using positive coping strategies

• Session 10: Problem solving

• Session 11: Medication

• Session 12: Planning for the future

Each weekly session was conducted with a group of 5–7 young women and lasted approximately 45 minutes. The session structure consisted of a recap of the previous session and feedback on how participants used the new technique, general information, topic related examples, group exercises and discussion, homework exercises and a session summary sheet. At the end of each session, participants interacted during a snack break which fostered a sense of group cohesion.

#### Internal pilot

The intervention was delivered to five participants and assessment measures completed at key time points. The observations and experiences of the researchers during administration of the assessment measures underpinned modifications to some of the wording on some questionnaires to ensure that participants would understand what was being asked of them. Response options and scoring categories were also simplified on some assessment measures. Following this phase of the study, the parents/guardians of participants were invited to attend a focus group to provide feedback on their experiences and to suggest modifications to the study. Parents suggested that a picture be included on each weekly session outline to aid participants in remembering and applying the technique discussed that week. It was also recommended that participants be provided with a summary sheet at the end of the programme. The young women involved were asked for their feedback and they suggested that a certificate of participation be presented at the end of the intervention programme.

### Measures

The primary outcome measures explored in this study were strategies used to cope with pain and pain management knowledge. Secondary outcome measures quantified pain severity, pain interference, pain self-efficacy and pain-catastrophizing. Following delivery of the intervention, qualitative evaluation was conducted with stakeholders including group participants, parents/guardians, teachers, principals and/or staff members at Adult Day Centres to evaluate the programme and its impact. Various measures were administered at different time points – these are summarised in Table 1.

**Table 1:** Outcome measures and time points

**Measure**	**Questionnaire**	**T1: Baseline/pre-intervention**	**T2: 5 weeks**	**T3: 10 weeks**	**T4: Post-intervention**	**T5: 12 week follow-up**
**Primary outcome measures**	Pain Coping Strategies	x			x	x
	Pain Coping Scenarios	x	x	x	x	x
	Pain Knowledge	x	x	x	x	x
**Secondary outcome measures**	Visual Analogue Scale [31]	x	x	x	x	x
	Modified version of the Brief Pain Inventory – Short Form [32]	x			x	x
**Process variables**	Modified version of the Self-Efficacy scale for child functioning despite chronic pain [33]	x			x	x
	Pain Catastrophizing Scale – Parent version (PCS-P) [23]	x			x	x
**Predictor variables**	Background Information Questionnaire	x				

#### Primary outcome measures

The Pain Coping Strategies Questionnaire [27] and the Pain Coping Scenarios Questionnaire (modified from McManus, [27]) were used to measure pain coping. These measures were previously compared pre-intervention, post-intervention and at 1-month follow-up, in a pilot study examining the feasibility and clinical utility of CBT for people with an intellectual disability [27]. On the Pain Coping Strategies Questionnaire, all participants showed an increase in wellness-focused coping strategies from pre-intervention to post-intervention assessment, however, these were generally not maintained at follow-up. On the Pain Coping Scenarios Questionnaire, 4 of the 5 participants showed an increase in wellness-focused coping strategies from pre-intervention to post-intervention assessment and this was maintained at follow-up for 3 participants.

On the Pain Coping Strategies Questionnaire, participants were asked to name all the different things they do to deal with their menstrual pain. Participant’s responses were coded as wellness-focused coping strategies or illness-focused coping strategies, based on the work of Jensen & Karoly [28]. Wellness-focused coping strategies included relaxation, exercise and distraction techniques. Illness-focused strategies included rest and use of medication. Coping strategies used were also coded as “behavioural” or “cognitive” in orientation, in line with the categorisation system used in the *Feeling Better* programme. The effectiveness of pain coping strategies was also assessed by asking participants to rate how well each of the strategies worked using three response options: “works very well”, “works sometimes”, “doesn’t work at all”. The Pain Coping Scenarios Questionnaire asked participants how they would cope with pain in four hypothetical situations i.e. during the night, at school/at their day programme, at home and during a social activity. This determined if participants could generalise techniques learnt during the intervention programme to situations in which they may experience menstrual pain. Again, coping strategies were coded as wellness-focused or illness-focused and as behavioural or cognitive in orientation. The Pain Knowledge Questionnaire [27] assessed knowledge of pain coping strategies using a seven item multiple choice questionnaire. Response options were “yes”, “no” and “don’t know”. The items on the scale reflect the core domains of the intervention e.g. relaxation, exercise, distraction, challenging negative thoughts. A clarifying question was asked following each correct answer (“Can you give me an example?”) to confirm that the participant had some knowledge related to their answer. If the participant answered the question correctly but the supplementary information provided demonstrated inaccuracies in knowledge, the response was not credited as correct.

The three primary outcome measures were administered at T1: baseline (pre-intervention), T4: 12 weeks from baseline (post-intervention) and T5: 3 months follow-up. Post intervention measures were administered by another researcher, independent of the research team, to minimise social desirability bias. The Pain Coping Scenarios Questionnaire and the Pain Knowledge Questionnaire were administered at two additional time points - T2: 5 weeks from baseline and T3: 10 weeks from baseline. These additional time point measures facilitated process evaluation to determine which elements of the intervention programme were most effective for this population.

#### Secondary outcome measures

Because variability in outcome measures across clinical trials hinders the evaluation of treatments, the Initiative on Methods, Measurement and Pain Assessment in Clinical Trials (IMMPACT) recommended that six core outcome domains should be considered when designing chronic pain clinical trials. These were defined as pain intensity, physical functioning, emotional functioning, participant ratings of improvement and satisfaction with treatment, symptoms and adverse events and participant disposition [29]. Pain, or pain intensity, is not the key target of the intervention in this study as the goal of CBT-based pain management programmes is not to reduce pain. Rather, it is to enhance adaptive coping and support the individual to resume a more productive and enjoyable life despite pain [29]. Lynch-Jordan et al. [30] demonstrated that the rate of change of functional disability was significantly more rapid than the change in pain intensity over the course of psychological treatment for children with chronic pain.

Participants rated pain intensity during their last menstrual period using a coloured Visual Analogue Scale (VAS) [31]. On this scale, 0 = no pain and 10 = lots of pain. Due to the challenges associated with using numerical rating scales with individuals with intellectual disabilities, proxy measures of pain severity were also obtained from the parents of participants. Pain interference was measured by a modified version of the Brief Pain Inventory – Short Form [32]. This questionnaire used a likert scale where 0 = did not interfere and 10 = completely interferes. The modified version of the Brief Pain Inventory – Short Form used in this study was specifically generated for the purpose of this research project. The specific modifications required included front and back female body outlines to enable participants to identify the areas of the body in which they experienced menstrual pain, omission of some questions e.g. questions regarding pain in the last 24 hours (participants may not be menstruating at the time of administration of questionnaires), changes to the questions on categories assessed (wording changes and additional categories included) and modifications to the response categories by including the use of a visual analogue rating scale in conjunction with a numerical rating scale. Modifications to the original Brief Pain Inventory – Short Form were required to enable it to be understood by and used with people with intellectual disabilities as well as to make it relevant to the assessment of menstrual pain, which is an intermittent rather than a constant type of pain.

Secondary outcome measures were administered to participants at T1: baseline (pre-intervention), T4:12 weeks from baseline (post-intervention) and T5: 3 months follow-up. Secondary outcome measures were administered to parents/guardians at T1, T4 and T5. The VAS was also administered to participants at T2: 5 weeks from baseline and T3: 10 weeks from baseline to determine the impact of the intervention on pain severity, over time.

## Supplementary research methodologies

### Moderator analyses

#### Process variables

Process variables are those which lead to change in the outcome measures. These included the level of cognitive ability of participants, pain self-efficacy and pain catastrophizing. Level of cognitive ability was confirmed by Team Leaders with reference to information recorded on the National Intellectual Disability Database (NIDD). The NIDD is a database of information about people who receive intellectual disability services in Ireland or who are in need of these services.

Pain self-efficacy refers to an individual’s belief that they can perform certain tasks related to school, friends and family even when they are in pain. It is an important variable to consider given its potential impact on participants’ willingness to implement strategies to cope with their pain. Participant pain self-efficacy was measured using a modified version of the self-efficacy scale for child functioning despite chronic pain [33], and was read to participants. Modifications included reducing the total number of items on the original scale and reducing the response options to three (1 = Always, 2 = Sometimes and 3 = Never).

Pain catastrophizing is a negative cognitive-affective response to anticipated or actual pain and has been consistently associated with pain intensity and pain related activity interference [34]. Pain catastrophizing was assessed using the parent version of the Pain Catastrophizing Scale (PCS-P) [23]. This is a thirteen item rating scale to assess parents’ thoughts and feelings when their child is in pain. The 5 response options are: not at all (disagree), mildly (agree), moderately (agree), severely (agree) and extremely (agree). Pain self-efficacy and pain-catastrophizing were assessed at the same time-points as the outcome variables i.e. T1: baseline (pre-intervention), T4:12 weeks from baseline (post-intervention) and T5: 3 months follow-up.

#### Predictor variables

There were a number of variables which may have moderated the impact of the outcome measures in this study. These included socio-demographics such as age, education etc.; time since onset of menstruation; frequency and duration of menstruation; number and frequency of menstrual symptoms experienced and history, treatment and use of medication to manage gynaecological problems and other medical conditions. Moderator analyses were conducted to examine the conditions under which moderating variables interacted with the intervention condition as predictor variables in the main effect analyses.

Pre- and post-intervention data collection has been completed. Data analysis is currently being conducted.

### Data analyses

#### Sample size and power calculation

There are a lack of well-conducted controlled trials and a lack of information about effect sizes of CBT with people with an intellectual disability within published research in this area. The authors were reliant on the few previous studies available regarding this area of research and for this reason, the feasibility study by Hassiotis et al. [35] was used as a guideline in calculating sample size requirement. This study proposed a total sample of 30 to be allocated across two conditions although there is currently discussion that feasibility trials ought to include up to 60 individuals. We based our sample size on the Hassiotis paper and allowed for 20% attrition, thus we recruited 36 participants across the treatment and control conditions, with n = 18 in each arm.

An *a priori* power analysis was conducted using G*Power [36]. With α = .05 (two-tailed), the proposed sample (*N* = 36) would detect moderate effects between groups (d = .50) with over 95% power using a repeated measures F test. A total sample of 16 participants would be needed to detect large effects (d = .8) with over 95% power using a repeated measures F test. For the regression analyses, a sample of 36 participants (α = .05) with three predictors would detect large effects (f^2^ = .35) with a power calculation of 80.95%, while six predictors would detect large effects with a power of 65.58%. The current sample was deemed appropriate as regression models with power less than 50% are deemed unreliable [37].

#### Quantitative analysis

Multivariate analysis of variance (MANOVA) will be used to examine differences in the primary and secondary outcome measures between the intervention and matched control groups. Within-groups differences will be measured at each time-point, including baseline. Baseline scores will be included as a covariate to allow for regression to the mean and a multiple regression model will be used. A step-wise method of regression analysis will be conducted with predictors entered using the backward method. This method will be used for exploratory model building and will be cross-validated by then randomly splitting the data. The dependent variables of interest will be strategies used to cope with pain and pain knowledge. The reliability of the Pain Coping Strategies Questionnaire and the Pain Coping Scenarios Questionnaire will be assessed by Cronbach’s alpha and validity will be assessed by correlating the measures against one other. Predictors will include the demographic characteristics of age and degree of intellectual disability as well as time since onset of menstruation; frequency and duration of menstruation; number and frequency of menstrual symptoms experienced and history, treatment and use of medication to manage gynaecological problems and other medical conditions.

Process evaluation will be conducted to assess change over time for the primary outcome measures. This will be done by looking at within groups differences at each of the assessment time points. To assist with process evaluation, sessions 2 – 5 focused on behavioural coping strategies and sessions 7 – 10 were dedicated to cognitive coping strategies.

#### Qualitative analysis

Following the intervention, group discussions with interested stakeholders e.g. group participants, parents/guardians, teachers and staff members were facilitated. The data was analysed using thematic analysis to enable the identification, analysis and reporting of themes in the data.

### Ethical approval

The research study protocol, participant information leaflets, consent forms and assessment measures were granted ethical approval by the Senate Research Ethics Committee of City University London on 16/5/2012 (Ref: PSYETH 11/12 026).

As research participants were recruited from the catchment area of the Brothers of Charity Services (an organisation which provides support services to individuals with intellectual disabilities in County Galway, Ireland), ethical approval was also sought from the organisations Research Ethics Committee. Ethical approval was granted by the Brothers of Charity Services Research Ethics Committee on 25/6/2012.

## Discussion

In this study, we evaluated the impact on pain coping and pain management knowledge, of a menstrual pain management programme for young women with intellectual disabilities. We expected participants in the intervention group to report the use of a greater number of coping strategies and have greater knowledge of pain management strategies at both T4 and T5, compared to control group participants.

The content of the menstrual pain management programme was adapted from the theory-based cognitive behavioural therapy programme “Feeling Better – A manual for carers working with people who have intellectual disabilities and chronic pain” [20]. The programme that was developed reflected the input from the parents/guardians of research participants, on both content and delivery methods. The PRP qualitative component ensured that the theory-based programme was specifically tailored to meet the needs of this population. As stakeholders, parents/guardians have insights into the training needs of their daughters and the possible challenges in delivering such a programme. Caregivers control access to medical/health services [3,38], so it is very important that they are involved with health interventions for those who are in their care.

A significant advantage to this study was the inclusion of process evaluation to determine which elements of a cognitive behavioural therapy programme work best for individuals with intellectual disabilities. Hunter [39] identified the need for clarification on effective components of CBT approaches for pre-menstrual symptoms and this is particularly relevant for this population. Moderator analyses of the outcome of the intervention will enable a better understanding of who this type of training is most effective with and under what conditions. Such information will enable us to optimize treatment for each individual into the future. For this reason, a number of variables which are assumed to be related to pain coping were measured in this study.

## Conclusion

This research study aimed to evaluate the efficacy of a menstrual pain management programme for young women with intellectual disabilities. If successful, this training could be incorporated within social, personal and health education (SPHE) initiatives delivered to young women with intellectual disabilities to enhance their adaptive coping skills and quality of life.

## Competing interests

The authors declare that they have no competing interests.

## Authors’ contributions

SK conceived of the study, participated in its design, is responsible for delivery of the intervention, and drafted the manuscript. SOH conducted the programme development focus group and collected outcome measures data. KS participated in the design of study, advised regarding statistical analysis and helped to draft the manuscript. CW participated in the design of the study and helped to draft the manuscript. BMcG participated in the design of the study and helped to draft the manuscript. All authors read and approved the final manuscript.

## Pre-publication history

The pre-publication history for this paper can be accessed here:

http://www.biomedcentral.com/1472-6874/14/107/prepub

## References

[B1] International Association for the Study of Pain International Association for the Study of Pain: classification of chronic pain Pain 1986 24 1 2263513094

[B2] Walsh M Morrison T McGuire BE Chronic pain in adults with an intellectual disability: prevalence, impact and health service use based on caregiver report Pain 2011 152 1951 1957 10.1016/j.pain.2011.02.031 21497999

[B3] McGuire BE Daly P Smyth F Chronic pain among people with an intellectual disability: Under-recognised and under-treated? J Intellect Disabil Res 2010 54 240 245 10.1111/j.1365-2788.2010.01254.x 20387264

[B4] McGuire BE Kennedy S Pain in people with an intellectual disability Curr Opin Psychiatry 2013 26 270 275 10.1097/YCO.0b013e32835fd74c 23508000

[B5] Davis AR Westhoff CL Primary dysmenorrhea in adolescent girls and treatment with oral contraceptives J Pediatr Adolesc Gynecol 2001 14 3 8 10.1016/S1083-3188(00)00076-0 11358700

[B6] Eden JA Hacker FN, Moore JG Dysmenorrhoea and premenstrual syndrome Essentials of Obstetrics and Gynaecology 1992 2 Philadelphia, PA: W.B. Saunders 332 337

[B7] Kyrkou M Health issues and quality of life in women with intellectual disability J Intellect Disabil Res 2005 49 10 770 772 10.1111/j.1365-2788.2005.00749.x 16162125

[B8] American Congress of Obstetricians and Gynaecologists Dysmenorrhea 2011 Patient Education Pamphlet

[B9] Tu CH Niddam DM Chao HT Liu RS Hwang RJ Yeh TC Hsieh JC Abnormal cerebral metabolism during menstrual pain in primary dysmenorrhea Neuroimage 2009 47 28 35 10.1016/j.neuroimage.2009.03.080 19362153

[B10] Tu CH Niddam DM Chao HT Chen YS Wu YT Yeh TC Lirng JF Hsieh JC Brain morphological changes associated with cyclic menstrual pain Pain 2010 150 462 468 10.1016/j.pain.2010.05.026 20705214

[B11] Tu CH Niddam DM Yeh TC Lirng JF Cheng CM Chou CC Chao HT Hsieh JC Menstrual pain is associated with rapid structural alterations in the brain Pain 2013 Epub, May 18 10.1016/j.pain.2013.05.022 23693160

[B12] Vincent K Warnaby C Stagg CJ Moore J Kennedy S Tracey I Dysmenorrhea is associated with central changes in otherwise healthy women Pain 2011 152 1966 1975 10.1016/j.pain.2011.03.029 21524851

[B13] Iacovides S Baker FC Avidon I Bentley A Women with dysmenorrhea are hypersensitive to experimental deep muscle pain across the menstrual cycle J Pain 2013 Epub Jun 12 10.1016/j.jpain.2013.04.010 23769507

[B14] Berkley KJ Primary Dysmenorrhea: An Urgent Mandate Pain 2013 XXI 3 1 8 Clinical Updates

[B15] Eccleston C Williams AC d C Morley S Psychological therapies for the management of chronic pain (excluding headache) in adults Cochrane Database Syst Rev 2008 4 Art. No.: CD007407 doi:10.1002/14651858.CD007407 10.1002/14651858.CD007407.pub2 19370688

[B16] Proctor ML Murphy PA Pattison HM Suckling J Farquhar CM Behavioural interventions for primary and secondary dysmenorrhoea. In Proctor, Michelle Cochrane Database Syst Rev 2007 3 CD002248 doi:10.1002/14651858.CD002248.pub3. PMID 17636702 17636702 10.1002/14651858.CD002248.pub3 PMC7137212

[B17] McCabe MP Gillivray JA Newton DC Effectiveness of treatment programmes for depression among adults with mild/moderate intellectual disability J Intellect Disabil Res 2006 50 239 247 10.1111/j.1365-2788.2005.00772.x 16507028

[B18] Lindsay WR Neilson C Laurensen H Stenfert-Kroese B, Dagnan D, Loumidis K Cognitive-behaviour therapy for anxiety in people with learning disabilities Cognitive behaviour therapy for people with learning disabilities 1997 London: Routledge 124 140

[B19] Willner P Jones J Tam R Green G A randomised controlled trial of a cognitive behavioural anger management group for clients with learning disabilities J Appl Res Intellect Disabil 2002 15 224 235 10.1046/j.1468-3148.2002.00121.x

[B20] McManus S McGuire BE Feeling Better – A manual for carers working with people who have intellectual disabilities and chronic pain 2010 Brighton: Pavilion Publishing Ltd

[B21] McManus S McGuire BE Cognitive behavioural therapy for chronic pain in people with an intellectual disability: a case series using components of the *Feeling Better* programme J Intellect Disabil Res 2014 58 3 296 306 doi:10.1111/jir.12018 10.1111/jir.12018 23387426

[B22] Sullivan MJL Thorn BE Haythornthwait J Keefe FJ Martin M Bradley L Lefebvre JC Theoretical perspectives on the relation between catastrophizing and pain Clin J Pain 2001 17 53 64 10.1097/00002508-200103000-00008 11289089

[B23] Goubert L Eccleston C Vervoort T Jordan A Crombez G Parental catastrophizing about their child’s pain. The parent version of the Pain Catastrophizing Scale (PCS-P): A preliminary validation Pain 2006 123 254 263 10.1016/j.pain.2006.02.035 16644128

[B24] Burkitt CC Breau LM Zabalia M Parental assessment of pain coping in individuals with intellectual and developmental disabilities Res Dev Disabil 2011 32 1564 1571 10.1016/j.ridd.2011.01.050 21377323

[B25] Craig P Dieppe P Macintyre S Michie S Irwin I Petticrew M Developing and evaluating complex interventions: the new Medical Research Council guidance Br Med J 2008 337 doi:10.1136/bmj:a1655 10.1136/bmj.a1655 PMC2769032 18824488

[B26] Rifkin SB Rapid Rural Appraisal: its use and value for health planners and managers Public Administration 1996 74 3 509 526 10.1111/j.1467-9299.1996.tb00882.x

[B27] McManus S Cognitive behaviour therapy for chronic pain among people with intellectual disability: a case series. D.Psych. Sc. (Clin Psych) Thesis 2007 National University of Ireland Galway, School of Psychology

[B28] Jensen MP Karoly P Turk DC, Melzack R Self-report scales and procedures for assessing pain in adults Handbook of Pain Assessment 1991 2 New York: Guilford Press 15 34

[B29] Turk DC Cognitive-behavioural perspective on chronic pain patients Reg Anesth Pain Med 2003 28 6 573 579 10.1097/00115550-200311000-00016 14634950

[B30] Lynch-Jordan AM Sil S Peugh J Cunningham N Kahikar-Zuck S Goldschneider KR Differential changes in functional disability and pain intensity over the course of psychological treatment for children with chronic pain Pain 2014 doi:10.1016/j.pain.2014.06.008 10.1016/j.pain.2014.06.008 PMC4467550 24954165

[B31] McGrath PA Seifert CE Speechley KN Booth JE Stitt L Gibson ME A new analogue scale for assessing children’s pain: an initial validation study Pain 1996 64 435 443 10.1016/0304-3959(95)00171-9 8783307

[B32] Cleeland CS Ryan KM Pain assessment: global use of the Brief Pain Inventory Ann Acad Med Singapore 1994 23 2 129 138 8080219

[B33] Bursch B Tsao JCI Meldrum M Zelter LJ Preliminary validation of a self-efficacy scale for child functioning despite chronic pain (child and parent versions) Pain 2006 125 1–2 35 42 16740360 10.1016/j.pain.2006.04.026 PMC2394279

[B34] Quartana PJ Campbell CM Edwards RR Pain catastrophizing: a critical review Expert Rev Neurother 2009 9 5 745 758 10.1586/ern.09.34 19402782 PMC2696024

[B35] Hassiotis A Serfaty M Azam K Strydom A Martin S Parkes C Blizard R King M Cognitive behaviour therapy (CBT) for anxiety and depression in adults with mild intellectual disabilities (ID): a pilot randomised controlled trial Trials 2011 12 95 101 10.1186/1745-6215-12-95 21492437 PMC3102627

[B36] Faul F Erdfelder E Lang AG Buchner A G*Power 3: A flexible statistical power analysis program for social, behavioural and biomedical sciences Behav Res Methods 2007 39 175 191 10.3758/BF03193146 17695343

[B37] Vacha-Haase T Thompson B How to estimate and interpret various effect sizes J Counsell Psychol 2004 51 4 473 481

[B38] McGuire BE Daly P Smyth F Lifestyle and health behaviours of adults with an intellectual disability J Intellect Disabil Res 2007 51 497 510 10.1111/j.1365-2788.2006.00915.x 17537163

[B39] Hunter MS Cognitive behavioural interventions for premenstrual and menopausal problems J Reprod Infant Psychol 2003 21 183 194 10.1080/0264683031000155006

